# The Right Inferior Frontal Gyrus Plays an Important Role in Unconscious Information Processing: Activation Likelihood Estimation Analysis Based on Functional Magnetic Resonance Imaging

**DOI:** 10.3389/fnins.2022.781099

**Published:** 2022-03-24

**Authors:** Jilong Shi, Haojie Huang, Ruichen Jiang, Xuechen Mao, Qin Huang, Anmin Li

**Affiliations:** ^1^School of Psychology, Shanghai University of Sport, Shanghai, China; ^2^Department of Physical Education, Xiamen University, Xiamen, China; ^3^School of Teacher Education, Anqing Normal University, Anqing, China

**Keywords:** unconsciousness, masked priming, activation likelihood estimation, right inferior frontal gyrus, priming effect

## Abstract

Unconsciousness is a kind of brain activity that occurs below the level of consciousness, and the masked priming paradigm is a classic paradigm to study unconscious perceptual processing. With the deepening of unconscious perception research, different researchers mostly use different experimental materials and different masked priming paradigms in a single experiment but not for the comprehensive analysis of the unconscious information processing mechanism itself. Thus, the purpose of this study is to conduct a comprehensive analysis through a cross-experimental paradigm, cross-experimental materials, and cross-experimental purposes. We used activation likelihood estimation to test functional magnetic resonance imaging studies, involving 361 subjects, 124 foci in eight studies representing direct comparison of unconscious processing with baseline, and 115 foci in 10 studies representing direct comparison of unconscious priming effects. In the comparison of unconscious processing and baseline, clusters formed in the left superior parietal gyrus, the right insular gyrus, and the right inferior frontal gyrus (IFG) triangular part after correcting for familywise error (FWE). In the comparison of priming effects, clusters formed in only the right IFG triangular part after correcting for FWE. Here, we found that ventral and dorsal pathways jointly regulate unconscious perceptual processes, but only the ventral pathway is involved in the regulation of unconscious priming effects. The IFG triangular part is involved in the regulation of unconscious perceptual processing and unconscious priming effects and may be an important brain area in unconscious information processing. These preliminary data provide conditions for further study of the neural correlation of unconscious information processing.

## Introduction

In general, consciousness includes all mental activities, such as human thoughts, feelings, cognition, and memory, while unconsciousness contains deeper mental processes that are not perceived by consciousness. Therefore, unconsciousness is the brain activity that occurs below the level of consciousness. In human behavior, a visual or facial message that flashes for tens of milliseconds can still have an impact on human consciousness. However, when a word, a figure, or a face is close to other visual stimuli in space and time, it becomes indistinguishable, even beyond human perception of the information. This is a visual phenomenon called masking (Dehaene et al., 2001). Evett and Humphreys (1981) first used the masked priming paradigm to measure unconscious perception. Their masked priming paradigm has four levels of structure: forward mask – priming stimulus – target stimulus – backward mask. However, this method controls the presentation time of the target stimulus and prime stimulus of 50 ms, which is prone to produce a fusion effect: the target stimulus and prime stimulus cannot be distinguished at all (Kouider and Dehaene, 2007).

With increased unconscious perception research, researchers have improved the four-level structure of the masked priming paradigm to improve its credibility (Greenwald et al., 1996). To date, the masked priming paradigm may be divided into three types: the forward masking paradigm, the backward masking paradigm, and the sandwich masking paradigm, including the forward and backward masking paradigms. The forward masked priming paradigm was proposed by Forster and Davis (1984) as a three-level structure because there is no backward masking. The target stimulus itself is seen as a very powerful masking, and this structure consists of forward masking, a short prime word (60 ms or below), and a target word. The principle of the backward masking paradigm is to reduce the visibility of the prime stimulus through the backward pattern mask so that the subjects can neither recognize the prime (McCauley et al., 1980) nor make the existence-absence judgment significantly more than the opportunity (Fowler et al., 1981; Balota, 1983; Marcel, 1983; Cheesman and Merikle, 1984). Since sandwich masking includes a forward mask and backward mask, it was considered to have a more effective masking effect (Greenwald et al., 1996; Gao and Zhang, 2014). However, regardless of the masking paradigm, the spatial and temporal presentation of the stimulus could be restricted to prevent the participants from consciously processing the stimulus cue.

In unconscious perception studies, another important concept is unconscious priming. Namely, “invisible” stimuli can nonetheless affect behavioral responses to subsequent probe stimuli presented shortly after the mask (Henson et al., 2008). For example, Greenwald et al. (1995) conducted a typical experiment in which the subjects were instructed to complete a classification task, where the targets were divided into positive (e.g., “happy”) or negative (e.g., “vomit”) words, and the front of these words had a congruent prime (i.e., a word from the same category, such as the “love” preceding the target “happy” or an incongruent prime “vomit” preceding “happy”). Subjects were faster in congruent trials than in incongruent trials (Greenwald et al., 1995). Therefore, the priming effect depends on whether the prime and the target categories are congruent or incongruent, which means that unconscious priming originates from competition between the prime stimulus category and the target stimulus category, thus reflecting categorical congruity (Kouider and Dehaene, 2007). Numerous functional magnetic resonance imaging (fMRI) studies have shown that the incongruent trial produced a larger increase in BOLD signals in brain regions than the congruent trial, although this was unconscious, possibly because the incongruent trial involved more cognitive resources (Dehaene et al., 2001; Nakamura et al., 2007; Kouider et al., 2010).

Numerous studies have used the masked priming paradigm to prove that people can process emotions, words, graphics, and other information presented at the unconscious level (Dehaene et al., 2001; D’Ostilio and Garraux, 2012a; Prochnow et al., 2013; Sato et al., 2016; Tacikowski et al., 2017). However, due to the differences in experimental purposes, different researchers mostly adopted different experimental materials and different masked priming paradigms for the study of a single experimental purpose and did not comprehensively analyze the unconscious information processing mechanism itself. With the application of fMRI technology in the study of unconscious information processing and the emergence of activation likelihood estimation (ALE) analysis methods (Brooks et al., 2012; Meneguzzo et al., 2014), it is possible to explore cross-experimental materials, cross-experimental paradigms, and cross-experimental purposes.

Most studies have accepted the view that the processing of cognitive information is mainly carried out by the ventral and dorsal pathways (Weiskrantz et al., 1974; Mishkin et al., 1983; Rees et al., 2002; Weiller et al., 2011). Previous studies have believed that the dorsal pathway is an unconscious perceptual process pathway that can directly connect sensory information and response parameters without consciousness (Neumann and Klotz, 1994). Although dorsal pathways can explain some simple unconscious motor processes, most cognitive activities cannot be summarized by a single visual pathway (Weiller et al., 2011). Therefore, researchers began to focus on the joint role of the ventral and dorsal pathways in unconscious perceptual processes (van Polanen and Davare, 2015). Ulrich and Kiefer (2016) used functional connectivity analysis to demonstrate that the ventral and dorsal pathways jointly regulate unconscious perceptual processes and pointed out that the two pathways mainly interact in the inferior occipital gyrus and posterior parietal lobe. However, it is well known that functional connectivity analysis relies on strong experimental hypotheses, and this study aimed to explore whether unconscious perceptual processes are mediated by both ventral and dorsal pathways at the whole brain level via ALE analysis.

In summary, this study aims to solve three key problems through cross-paradigm, cross-material, and cross-purpose experiments. First, we explored whether we can find the key brain regions specific to unconscious perceptual processes and unconscious priming effects in the masked priming paradigm. Second, we examined whether unconscious perceptual processes are regulated by both ventral and dorsal pathways. Finally, we examined whether the priming effect needs more brain regions to be involved because of the longer response time in incongruent compared to neutral or congruent conditions.

## Materials and Methods

### Study Selection

We selected studies through a standard search in the PubMed, Web of Science, and Baidu Scholar databases using the terms (fMRI or MRI) AND (unconscious or subliminal or subconscious) AND (mask or prime). To be included in our meta-analysis, studies met the following criteria: (a) studies were published within the last two decades, between January 2001 and August 2021, (b) published in a peer-reviewed journal, (c) masked prime paradigms were used and the study included a direct contrast between brain activation to unconscious processing and baseline or a direct contrast between brain activation to incongruent and neutral or congruent conditions, (d) priming stimulus less than 50 ms, (e) were original articles written in English, (f) used fMRI and no other brain imaging modalities so that the data could be better aggregated for meta-analysis, and (g) reported the neural activation coordinates in Montreal Neurological Institute (MNI) or Talairach space. We excluded the results from patients and data from conditions focusing on pharmacological manipulation, and only healthy subjects were included (Eickhoff et al., 2009).

### Selected Studies

We retrieved 135 studies, and all studies used the same search coverage, but 86 studies did not meet our inclusion criteria. Of these 49 eligible studies, eight were not included in the meta-analyses because they did not provide Talairach or MNI peak activation coordinates. Of the 41 studies to date, only 24 studies specifically analyzed unconscious processing compared to baseline (resting state) or incongruent versus neutral or congruent conditions. Of the remaining 24 studies, eight used ROI analysis, which was excluded because they used exclusive ROI analysis, a technique that analyses only small regions of the brain based on *a priori* hypotheses. This procedure is different from whole brain analysis, which statistically analyses the activation of the entire brain in one analysis (Meneguzzo et al., 2014). Thus, this search left 16 whole-brain fMRI studies that included the comparison of unconscious processing to baseline or incongruent versus neutral or congruent conditions using the masked priming paradigm. Six of these studies directly compared the activation of brain regions between unconscious processing and baseline, eight directly compared the activation of brain regions in incongruent and neutral or incongruent and congruent conditions, and the other two included two experimental comparisons. All included studies are shown in Table 1.

**TABLE 1:** List of studies included in the ALE meta-analyses.

Study name	Subjects			Masked priming paradigm	Contrast	Foci	Statistical threshold
	Total	M/F	Age (SD)				
**(a) Unconscious activation greater than baseline**							
Dehaene et al., 2001	15	3/12	23.3	sandwich masking	semantic induction processing > baseline	6	voxel wise, *p* < 0.001; cluster-level corrected, *p* = 0.05
D’Ostilio and Garraux, 2012a ^#^	24	18/6	21 (2)	backward masking	incompatible, neutral, compatible > baseline	16	*p* < 0.001, uncorrected
Murawski et al., 2012	13	7/6	21.2	sandwich masking	temporal discounting > baseline	13	*p* < 0.05, FWE corrected
Prochnow et al., 2013	12	5/7	23.8 (3)	backward masking	subliminal faces > baseline	11	*p* < 0.005, uncorrected
Pichon et al., 2016 ^#^	30	15/15		sandwich masking	face recognition processing > baseline	7	*p* < 0.001, uncorrected
Sato et al., 2016	27	24/3	25 (4.6)	backward masking	averted and straight eyes > baseline	9	*p* < 0.005, uncorrected
Binder et al., 2017	30	10/20	21.94 (2.96)	backward masking	high level processing (PAS 1) >baseline	10	*p* < 0.05, FWE corrected
					high level processing (PAS 2) >baseline	9	
					low level processing (PAS 1) >baseline	2	
					low level processing (PAS 2) >baseline	21	
Tacikowski et al., 2017	26	15/11	25 (4)	sandwich masking	self-unaware > baseline	8	*p* < 0.001, uncorrected
					perceptual-unaware > baseline	12	
**(b) Incongruent activation greater than neutral and congruent activation**							
Luo et al., 2004	9	5/4		sandwich masking	semantic induction processing: incongruent > congruent	7	*p* < 0.01, uncorrected
Nakamura et al., 2010	24	16/8		forward mask	semantic induction processing: incongruent > congruent	6	*p* < 0.001, uncorrected
D’Ostilio and Garraux, 2012a ^#^	24	18/6	21 (2)	backward masking	perceptual induction processing: incompatible > compatible	18	*p* < 0.001, uncorrected
					perceptual induction processing: incompatible > neutral	9	*p* < 0.001, uncorrected
D’Ostilio et al., 2012b	26	11/15	22 (2)	backward masking	perceptual induction processing: incongruent > congruent	5	*p* < 0.05, FWE corrected
De Pisapia et al., 2012	20	10/10	24.8	backward masking	perceptual induction processing: incongruent > neutral	8	*p* < 0.05, FDR
Yang et al., 2012	27	14/13	22.45 (1.78)	backward masking	face recognition processing: incongruent > congruent	13	*p* < 0.001, Monte Carlo corrected
Ulrich et al., 2014	23	10/13	21.1 (2.6)	sandwich masking	semantic induction processing: incongruent > congruent	24	*p* < 0.001, uncorrected
Jiang et al., 2016	24	14/10	21.00 (1.54)	sandwich masking	semantic induction processing: incongruent > congruent	5	*p* < 0.001, AlphaSim corrected
Pichon et al., 2016 ^#^	30	15/15		sandwich masking	face recognition processing: gender incongruent > congruent	8	*p* < 0.001, uncorrected
Ulrich and Kiefer, 2016	31	15/16	24.5 (4.8)	sandwich masking	perceptual induction processing: incongruent > congruent	12	*p* < 0.05, FWE corrected

*^#^Denotes those two experimental comparisons are included.*

### Definition of the Unconscious Masked Priming Paradigm

For unconscious perceptual processes, the most common measurement is the masked priming paradigm, which involves a related and effectively masked priming before or after a highly visible target stimulus, and the target stimulus is more effectively processed than an unrelated prime. Studies included in this meta-analysis included perceptual induction processing, semantic induction processing, and face recognition processing using the masked priming paradigm (Kouider and Dehaene, 2007).

Kouider and Dupoux (2001) assessed the prime awareness of several prime durations and showed that a prime is considered truly invisible only if it lasts less than 50 ms. Therefore, unconscious presentation is most often achieved by a brief stimulus onset asynchrony usually not more than 50 ms (Brooks et al., 2012; Meneguzzo et al., 2014; Opstal, 2021). According to the discussion of many scholars on the presentation time of the masked priming paradigm prime stimulus, this meta-analysis excluded studies whose prime duration exceeded 50 ms.

### Quantitative Data Synthesis: Activation Likelihood Estimation

To examine unconscious activation and priming effects using the masked priming paradigm, we conducted two separate meta-analyses using BrainMap GingerALE version 3.0.2 software (Eickhoff et al., 2009). The ALE method is a voxelwise technique that provides information from convergence in the spatial location of the neural correlates across studies. Neural correlates, or foci from included studies, become the “activation likelihood” for each voxel in the brain, and for each voxel, ALE gives a score using a three-dimensional Gaussian probability density function to estimate the number of subjects in each study. The Gaussian distributions are then summed across studies to generate a map that estimates the likelihood of activation for each voxel (Laird et al., 2011; Turkeltaub et al., 2012). We applied the updated version of the ALE approach to conduct the meta-analyses using MNI coordinate “foci” from neuroimaging results and converting Talairach coordinates into MNI (SPM) for the analysis using GingerALE software (Eickhoff et al., 2010). The statistical results are presented in two thresholds. First, a familywise error (FWE)-corrected (Nichols and Hayasaka, 2003) threshold of *p* < 0.05 at the cluster level (cluster-forming threshold: *p* < 0.001 at the voxel level) through 1000 permutation tests, and then, an uncorrected threshold (*p* < 0.001, minimum cluster volume >200mm^3^). The reason why two statistical thresholds are used is to test the difference between different thresholds to obtain more reliable experimental results, We used an anatomical image overlay program called Mango (Creators, Jack Lancaster, Michael Martinez)^1^ and BrainNet Viewer (Creators, Mingrui Xia)^2^ to illustrate the results of our meta-analyses.

## Results

### Meta-Analysis One: Unconscious Perceptual Processing (Unconscious Processing > Baseline)

**FIGURE 1:**
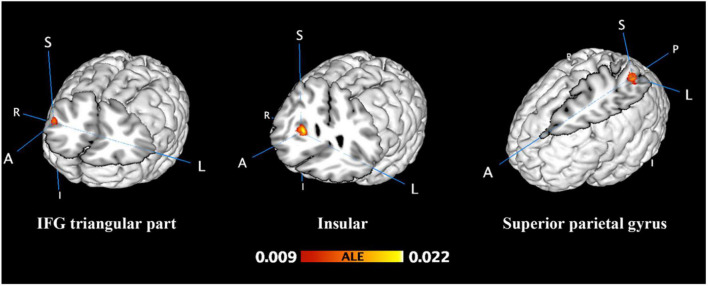
Unconscious activation greater than baseline. FWE-corrected at the cluster level (*p* < 0.05) with a cluster-forming threshold of *p* < 0.001.

**FIGURE 2:**
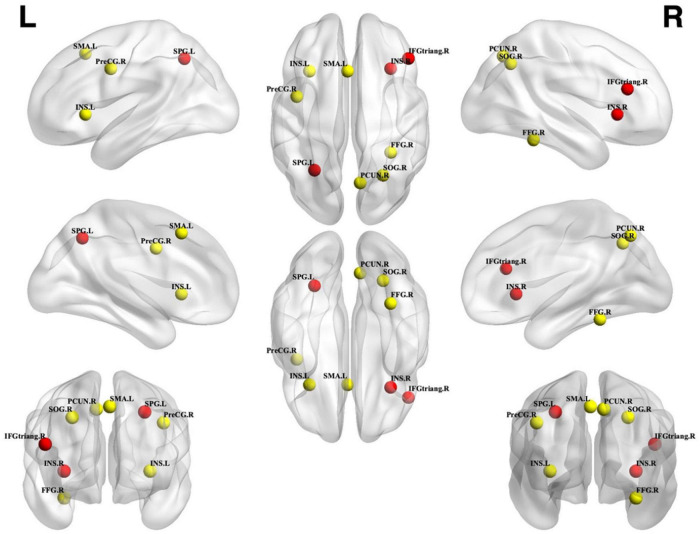
Unconscious perceptual processing. Unconscious activation greater than baseline. Spheres with a radius of 5 mm are visualized to present the statistical results of clusters formed. Red spheres represent results that survived FWE correction, while yellow spheres represent results that are uncorrected. SPG, Superior parietal gyrus; INS, Insular; IFGtriang, Inferior frontal gyrus triangular part; SMA, Supplementary motor area; PreCG, Precentral gyrus; SOG, Superior occipital gyrus; FFG, Fusiform gyrus; PCUN, Precuneus; L, left; R, right.

From 124 foci, 177 subjects and 12 separate experiments, 12 significant clusters were found that survived the cluster-level inference threshold (*p* < 0.001, uncorrected), of which three significant clusters survived the statistically more rigorous FWE-corrected analysis (*p* < 0.05). Cluster one was found in the left superior parietal gyrus (SPG) in BA7 (*x* = −28, *y* = −58, *z* = 44), cluster two was found in the right insula in BA47 (*x* = 32, *y* = 22, *z* = 0), and cluster three was found in the right inferior frontal gyrus (IFG) triangular part in BA45 (*x* = 46, *y* = 30, *z* = 20) (see Figures 1, 2 and Table 2).

**TABLE 2:** Unconscious activation greater than baseline.

Region	Side	BA	Cluster size in voxels	MNI coordinates			ALE	*Z* score
Superior parietal gyrus*	L	BA7	1112	−28	−58	44	0.017	4.61
Insular*	R	BA47	1048	32	22	0	0.021	5.26
Inferior frontal gyrus triangular part*	R	BA45	696	46	30	20	0.015	4.15
Insula	L	BA47	608	−32	20	0	0.018	4.64
Supplementary motor area	L	BA8	600	−2	20	48	0.013	3.80
Precentral gyrus	L	BA6	448	−42	0	36	0.012	3.61
Superior occipital gyrus	R	BA7	304	26	−62	40	0.013	3.73
Fusiform gyrus	R	BA37	208	32	−44	−20	0.013	3.76
Precuneus	R	BA7	208	8	−68	46	0.013	3.75

*Denotes FWE-corrected p-value at the cluster level (p < 0.05) with a cluster-forming threshold of p < 0.001. FWE, familywise error; L, left; R, right; MNI, Montreal Neurological Institute.

### Meta-Analysis Two: Unconscious Priming Effects (Incongruent > Neutral and Congruent)

**FIGURE 3:**
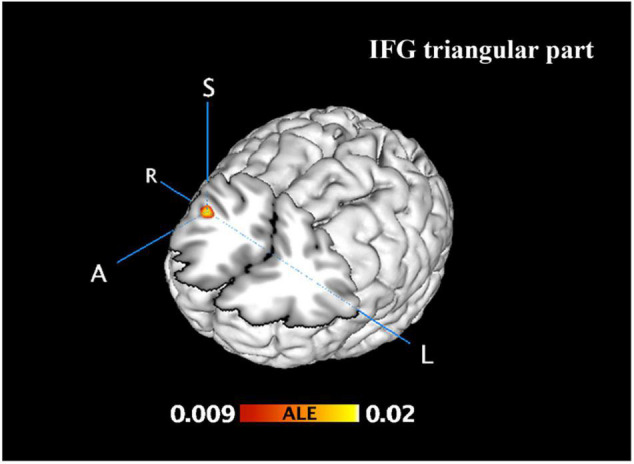
Incongruent activation was greater than neutral and congruent activation. FWE correction at the cluster level (*p* < 0.05) with a cluster-forming threshold of *p* < 0.001.

**FIGURE 4:**
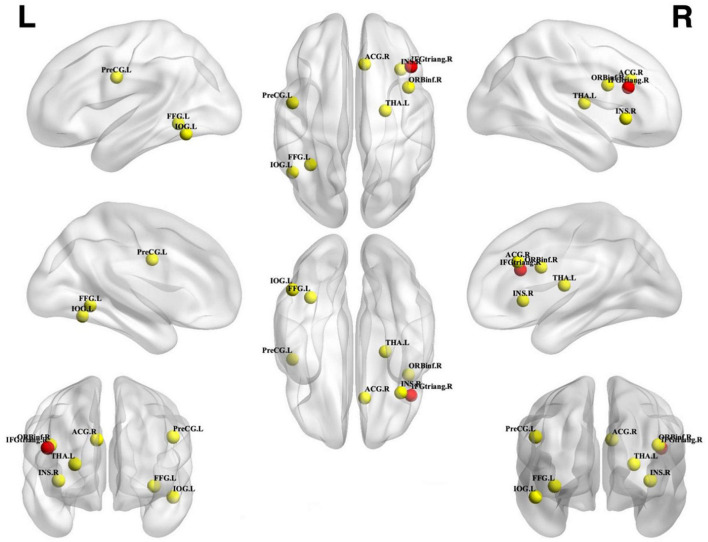
Unconscious priming effect. Incongruent activation was greater than congruent and neutral activation. Spheres with a radius of 5 mm are visualized to present the statistical results of clusters formed. Red spheres represent results that survived FWE correction, while yellow spheres represent results that are uncorrected. IFGtriang, inferior frontal gyrus triangular part; FFG, fusiform gyrus; ACG, anterior cingulate gyrus; THA, thalamus; IOG, inferior occipital gyrus; PreCG, precentral gyrus; INS, insular; ORBinf, inferior frontal gyrus opercular part; L, left; R, right.

From 115 foci, 238 subjects and 11 separate experiments, 10 significant clusters were found that survived the cluster-level inference threshold (*p* < 0.001, uncorrected), of which one significant cluster survived the statistically more rigorous FWE correction (*p* < 0.05). Cluster one was found in the right IFG triangular part in BA48 (*x* = 44, *y* = 24, *z* = 22) (see Figures 3, 4 and Table 3).

**TABLE 3:** Incongruent activation greater than neutral and congruent activation.

Region	Side	BA	Cluster size in voxels	MNI coordinates			ALE	*Z* score
Inferior frontal gyrus triangular part*	R	BA48	768	44	24	22	0.019	4.78
Fusiform gyrus	L	BA37	648	−34	−52	−6	0.018	4.71
Anterior cingulate gyrus	R	BA32	640	8	26	28	0.020	4.93
Thalamus	R		472	24	−10	10	0.015	4.14
Inferior occipital gyrus	L	BA37	384	−48	−58	−14	0.013	3.72
Precentral gyrus	L	BA4	360	−48	−4	30	0.015	4.05
Insula	R	BA47	248	36	22	−2	0.012	3.54
Inferior frontal gyrus opercular part	R	BA48	208	42	8	24	0.013	3.76

*Denotes FWE correction at the cluster level (p < 0.05) with a cluster-forming threshold of p < 0.001. FWE, familywise error; L, left; R, right; MNI, Montreal Neurological Institute.

## Discussion

We conducted a preliminary meta-analysis of fMRI studies comparing the activation of unconscious processing versus baseline and unconscious priming effects in the masked priming paradigm. In the unconscious condition, the prime stimulus and the target stimulus have longer latency periods in the incongruent condition than in the neutral and congruent conditions. We considered two studies that indicated differences in activation between incongruent and neutral conditions, since we assumed that incongruent conditions invoked more cognitive resources than both neutral and congruent conditions. However, caution must be taken in interpreting these findings in terms of categories and differences in visual stimuli, as they may influence the observed data (Meneguzzo et al., 2014). It is worth noting that, in consideration of the caution of interpreting cross-task research, we adopted a strict literature screening method in this meta-analysis in an effort to obtain more reliable research results. In fact, Brooks et al. (2012) conducted an ALE analysis based on fMRI for subliminal stimuli for the first time, but their study focused more on the brain activity level induced by different types of subliminal stimuli. However, Meneguzzo et al. (2014) also conducted an ALE analysis based on fMRI, which paid more attention to the differences in brain activities induced by supraliminal and subliminal stimuli. Since neither of these two studies only targeted at the unconscious masked priming paradigm, this study is also the first study to reveal the unconscious information processing mechanism underlying the masked priming paradigm through an ALE meta-analysis based on fMRI. The results of the meta-analysis also provided sufficient evidence for the three questions we planned to answer. Surprisingly, the right IFG triangular part plays a crucial role in unconscious information processing. In the following section, we will discuss the latest findings in this study.

The bilateral IFG is a heterogeneous region in two pathways of cognitive information processing, with the opercular part (BA44) connected mainly via the dorsal pathway, and the triangular part (BA45) and BA47 are mainly connected via the ventral pathway (Petrides and Pandya, 2007, 2009). Although the involvement of the bilateral IFG in the semantic process at the lexical level is unclear and controversial (Bookheimer, 2002), the triangular part (BA45) and orbital part (BA47) are related to the relevant features of semantic knowledge (Thompson-Schill et al., 1997). There is neuropsychological evidence that this region is involved in the control of semantic processing (Sharp et al., 2010). In the results of this meta-study, clusters were found in the IFG triangular part not only in the comparison between unconscious processing and baseline but also, more importantly, in the analysis of unconscious priming effects. In the case of incongruent processing, an unconscious perceptual process not only has a longer latency, but when considering that incongruent processing has been shown to activate more IFG regions than congruent and neutral processing (Ulrich et al., 2014; Jiang et al., 2016), this coincidence seems to provide evidence that an unconscious perceptual process can reach the semantic level. However, Ulrich et al. (2014) found in an analysis of the interaction between unconscious perceptual induction processing and semantic association that compared with perceptual induction processing, semantic induction processing had greater activations in the bilateral IFG (BA45) and the boundary part of the left anterior insula. It was further confirmed that the unconscious semantic priming effect only occurs after semantic induction processing but not after perceptual induction processing.

However, why is it the right IFG, not the left IFG, that shows the activation likelihood? It is well known that the left IFG triangular part is part of Broca’s area and is an important motor speech center (Thompson-Schill et al., 1997; Tremblay and Dick, 2016). Jackson (2021) conducted a meta-analysis of the neural correlativity of semantic control and found that semantic control depended on a distributed network composed of the IFG, post middle temporal gyrus, post inferior temporal gyrus, and dorsomedial prefrontal cortex. This network is left-dominant, with the left IFG more involved than the right IFG, and produces the strongest activation likelihood in the left IFG triangular part (Gonzalez et al., 2019; Jackson, 2021). However, although the left IFG generated more activations than the right IFG in the semantic control network, activations were still detected in the right IFG, and the role of the right IFG triangular part in semantic control remains unknown. Thus, when considering the mechanism of unconscious information processing in this study, the right IFG triangular part is likely to be a specific brain region for unconscious information processing in the masked priming paradigm. The ventrolateral prefrontal cortex (VLPFC) is known for its cognitive control functions (Miller and Cohen, 2001; Niendam et al., 2012) and plays an important role in the learning, retrieval, and maintenance of stimulate-response (S-R) rules (Toni and Passingham, 1999; Murray et al., 2000; Toni et al., 2001; Bunge et al., 2002, 2005; Crone et al., 2006), and the right IFG triangular part is a part of the VLPFC. On the one hand, in congruent trials, the relevant S-R rules and related responses have been preactivated by the prime, and the right IFG triangular part regulates response consistency in the congruent condition. On the other hand, in incongruent trials, priming activated inappropriate S-R mapping, and it is necessary to retrieve the appropriate S-R rules for the target response in a more detailed way (Ulrich and Kiefer, 2016). However, it has been shown that the IFG is recruited when competitive task representations are simultaneously activated (Bunge et al., 2002; Zhang et al., 2004; Badre et al., 2005; Moss et al., 2005; Souza et al., 2009). Thus, during incongruent trials, when considering that the prime preactivated an S-R rule inappropriate for the upcoming target, the triangular part may be involved in resolving the conflict (Ulrich and Kiefer, 2016).

In general, the dorsal (occipito – parietal) pathway is used for spatial perception (Mishkin et al., 1983; Rees et al., 2002), which is often called the “where” pathway. It has the function of “action vision” and provides strong input for the motor system to guide action (Michael et al., 2013). In this meta-analysis, we found that clusters formed in the dorsal pathway, such as the left SPG, the right superior occipital gyrus, and the right precuneus. As mentioned above, previous studies have suggested that dorsal pathways can explain some simple unconscious motor processing. Therefore, our results are consistent with previous studies (Neumann and Klotz, 1994). However, in information processing, the IFG is a heterogeneous area, and its triangular part is connected with the ventral pathway. The ventral (occipito-temporal) pathway is used for object perception and recognition (Mishkin et al., 1983; Rees et al., 2002). It is often called the “what” pathway and has the function of “recognizing vision” (Michael et al., 2013). Most studies suggest that the ventral pathway is a conscious processing pathway (Weiller et al., 2011; van Polanen and Davare, 2015). This meta-analysis found that the ventral pathway was involved in unconscious perceptual processing in the masked priming paradigm, such as the bilateral insula, right IFG triangular part, and right fusiform clusters. Although some studies have also shown that the ventral pathway participates in unconscious perceptual processes, their studies are based on the analysis of a single experimental purpose and a single experimental paradigm (Dehaene et al., 1998, 2001; Ulrich and Kiefer, 2016). In this study, ALE analysis provided strong evidence for the involvement of the ventral pathway in the regulation of unconscious perceptual processes from cross-purpose, cross-paradigm, and cross-material evidence. However, for most functions, both pathways are not mutually exclusive but rather work in parallel (Makris and Pandya, 2009; Rauschecker and Scott, 2009), constituting a loop that has to be passed at least once (Weiller et al., 2011). This loop may explain our findings that the two pathways cooperate to complete unconscious perceptual processing in the masked priming paradigm. Moreover, van Polanen and Davare (2015) proposed that the dorsolateral prefrontal cortex regulates the interaction between ventral and dorsal pathways so that the grasping action is completed. Thus, considering that the IFG is a heterogeneous region, we have reason to speculate that the interaction of the two pathways is mediated by the IFG in unconscious perceptual processing. The supplementary motor area and the precentral gyrus may participate in the preparation, planning, and execution of unconscious motor responses (Hanakawa et al., 2003; Witt et al., 2008).

However, in the regulation of the priming effect, we have different findings. Consistent with previous studies, our meta-analysis also found that the ventral pathway regulated the unconscious priming effect, such as the formation of clusters in the right IFG triangular part, the left fusiform gyrus and the left inferior occipital gyrus, but no significant clusters were found in the dorsal pathway. Moreover, to the best of our knowledge, only a few fMRI studies have shown that the unconscious prime effect involves the dorsal pathway (Aron et al., 2003; D’Ostilio and Garraux, 2012a; D’Ostilio et al., 2013). However, due to the use of arrow stimulation as a prime and target, the explanation of the priming effect is limited. Thus, there is no sufficient evidence to show that the two pathways coordinate in the unconscious priming effect in the masked priming paradigm (Ulrich and Kiefer, 2016).

The other two findings of this study are also critical. One is that the SPG formed clusters. The general theory holds that unconscious perceptual processes do not involve attention resources, and the appearance of SPG clusters challenges this view. The SPG receives attentional modulation signals from the prefrontal cortex and then applies appropriate attentional control to task-related sensory brain regions (Ulrich et al., 2013). Top–down attention control is not only a feature of spatial position and visual object but can also use the same neural mechanism for semantic information (Kiefer, 2008; Hoenig and Scheef, 2009; Grandjean et al., 2012). Thus, the discovery of SPG suggests that attention resources are involved in unconscious perceptual processes. Although SPG was found to form clusters across experiments, considering that different experimental designs have different control over conscious intervention, this result should be interpreted carefully. Another finding is that the insula forms clusters in unconscious perceptual processes and priming effects. Although the insula is known to be sensitive to both conscious and unconscious visual stimuli (Salomon et al., 2016), few studies have focused on the effect of claustrum hiding in the insula on unconscious information processing. The claustrum is hidden under the general area of the insula, which is thought to act as an on-off switch for consciousness (Crick and Koch, 2005; Brooks et al., 2012; Gattass et al., 2014; Meneguzzo et al., 2014; Chau et al., 2015). Whether the clusters formed in the insula in this study extend to the claustrum remains to be considered. However, there is a two-way connection between the claustrum and almost all areas of the cerebral cortex (Crick and Koch, 2005). Considering its bidirectional connectivity, whether or not claustrum may connect bilateral pathways and modulate unconscious perceptual processes is still a question worthy of more researchers’ attention.

## Conclusion

This study preliminarily confirmed our hypothesis. We found that ventral and dorsal pathways jointly regulate unconscious perceptual processes, but only the ventral pathway is involved in the regulation of unconscious priming effects. The right IFG triangular part is the key brain regions in unconscious information processing in the masked priming paradigm, which is involved in the regulation of unconscious perceptual processes and priming effects. These preliminary data provide the conditions for further investigation of the neural correlates of unconscious information processing.

## Data Availability Statement

The original contributions presented in the study are included in the article/supplementary material, further inquiries can be directed to the corresponding author.

## Author Contributions

All authors listed have made a substantial, direct, and intellectual contribution to the work, and approved it for publication.

## Conflict of Interest

The authors declare that the research was conducted in the absence of any commercial or financial relationships that could be construed as a potential conflict of interest.

## Publisher’s Note

All claims expressed in this article are solely those of the authors and do not necessarily represent those of their affiliated organizations, or those of the publisher, the editors and the reviewers. Any product that may be evaluated in this article, or claim that may be made by its manufacturer, is not guaranteed or endorsed by the publisher.

## Footnotes

1
http://ric.uthscsa.edu/mango

2
http://helab.bnu.edu.cn/brainnet-viewer
